# Frailty and healthcare utilisation across care settings among community-dwelling older adults in Singapore

**DOI:** 10.1186/s12877-020-01800-8

**Published:** 2020-10-06

**Authors:** Lixia Ge, Chun Wei Yap, Bee Hoon Heng, Woan Shin Tan

**Affiliations:** 1 Health Services & Outcomes Research, National Healthcare Group Pte Ltd, 3 Fusionopolis Link, #03-08 Nexus@one-north (South Lobby), Singapore, 13854 Singapore; 2 Geriatric Education and Research Institute, Singapore, Singapore

**Keywords:** Frailty, Healthcare utilisation, Community-dwelling older adults

## Abstract

**Background:**

Frailty is frequently found to be associated with increased healthcare utilisation in western countries, but little is known in Asian population. This study was conducted to investigate the association between frailty and healthcare utilisation in different care settings among community-dwelling older adults in Singapore.

**Methods:**

Data from a population health survey among community-dwelling adults were linked with an administrative database to retrieve data of healthcare utilisation (including government primary care clinic visits, specialised outpatient clinic visits, emergency department visits, day surgery and hospitalisations) occurred during a six-month look-back period and six-month post-baseline respectively. Baseline frailty status was measured using the five-item FRAIL scale, which was categorised into three groups: robust (0), pre-frail (1–2), and frail (3–5). Negative binomial regression was applied to examine the association between frailty with respective healthcare utilisation (dependent variables), controlling for other confounding variables.

**Results:**

In our sample of 701 older adults, 64.8% were of robust health, 27.7% were pre-frail, and 7.6% were frail. Compared to the robust group, frail individuals had a higher rate of specialised outpatient clinic visits (incidence rate ratio (IRR): 2.8, 95% confidence interval (CI): 1.2–6.5), emergency department visits (IRR: 3.1, 95%CI: 1.1–8.1), day surgery attendances (IRR: 6.4, 95%CI: 1.3–30.9), and hospitalisations (IRR: 6.7, 95%CI: 2.1–21.1) in the six-month period prior to the baseline and in subsequent 6 months (IRR: 3.3, 95%CI: 1.6–7.1; 6.4, 2.4–17.2; 5.8, 1.3–25.8; 13.1, 4.9–35.0; respectively), controlling for covariates.

**Conclusions:**

Frailty was positively associated with the number of specialised outpatient clinic visits, emergency department visits, day surgeries and hospitalisations occurred during 6 months prior to and after the baseline. As frailty is a potentially reversible health state with early screening and intervention, providing preventive activities that delay the onset or progression of frailty should have potential effect on delaying secondary and tertiary care utilisation.

## Background

Frailty can be defined as ‘a state of vulnerability to adverse outcomes resulting from the accumulation of deficits associated with clinical effects’ [1], and has been shown to be a common phenomenon among older adults [2]. A recent systematic review that gathered data from 21 studies and over 61,500 community-dwelling older adults found that the overall weighted average prevalence of frailty was 10.7% (range: 4.0–59.1%) [2]. With the absolute number of people aged 60 years and over expected to reach 2 billion in 2050 [3], the burden of frailty will increase [4].

In Singapore, 21.4% of the total population consisted of individuals aged 60 years and above in 2019 [5], which is projected to reach 40% by year 2050 [6]. Such drastic rise in both the number and proportion of older population inevitably translate into a surge in the number and proportion of frail individuals [7], which brings various challenges to health care and healthcare delivery as they are recognised as intensive users of health care services [8]. To forge a frailty-ready healthcare system, Singapore has re-organised its public healthcare system from six regional healthcare systems (RHS) into three integrated clusters to allow each cluster to have a fuller range of assets, capabilities, services and networks across different care settings to meet the challenges of population ageing and further care needs [9]. Each RHS is responsible for care integration and providing care to the population in a specific geographical region. Innovative projects have been implemented in each RHS to address the needs of the frail elderly in Singapore [10] but a deeper understanding of the frail older population and their patterns of healthcare utilisation is necessary for better resource planning and intervention prioritisation in public healthcare.

Based on self-reported incidence of healthcare resource utilisation, cross-sectional studies in international literature have found frailty to be associated with an increased likelihood of general practice (adjusted odds ratio (OR): 2.1–4.4), specialist (OR: 1.3–1.8), emergency department (OR: 2.5–6.2) and inpatient (OR: 2.1–3.3) service utilisation [8, 11–13]. These findings were also supported by the results from prospective cohort studies [14–16] and panel studies [17]. It is recogonised that most studies investigating the associations between frailty and healthcare utilisation were conducted in North American and European countries. As healthcare systems and access to care varies across countries, examining the association between frailty and healthcare utilisation using Singapore data will provide insights in an Asian setting where health seeking behaviours and utilisation patterns could differ.

As the assessment of frailty are typically determined based on the actual or estimated status of the person at the point of assessment, it usually does not account for the presence of acute conditions which might sway the determination of frailty status [18]. This will contribute to the variation in the magnitude of association between frailty and healthcare utilisation in different settings. While individuals who have been frail over a period may have persistently higher healthcare utilisation during retrospective and prospective observation periods, those with frailty caused by transient conditions might only have temporarily higher healthcare utilisation for a short period of time. In prior studies, the association between frailty and healthcare utilisation was explored using either retrospective or prospective data in different population. There is a scarcity of research that examined their associations using both retrospective and prospective utilisation data. As such, little is known about how the magnitude of association differs in different periods of time. This study aims to investigate the association of frailty and healthcare utilisation in community-dwelling older adults aged 60 years and above, with utilisation data collected in two different periods of time: 1) 6 months prior to the baseline frailty assessment, and 2) 6 months after the baseline assessment.

## Methods

### Study participants

Older adults aged 60 years and over (*n* = 701) who agreed to use their National Registration Identity Card (NRIC) number to link with administrative database to retrieve healthcare utilisation data were sampled from the Population Health Index (PHI) study, a population-based health survey conducted in the Central Region of Singapore. The baseline data of PHI which were collected during November 2015 to November 2016 were used for this study. The sampling procedure and survey methodology of the baseline PHI study has been described elsewhere [19–21]. In brief, eligible participants (Singapore citizens or permanent residents, aged 21 years and above and lived in the selected housing unit for the past 6 months) staying in randomly selected household units in the Central Region of Singapore were identified via door-to-door visits by trained surveyors and one eligible household member was randomly selected using Kish grid [22]. There were 1942 eligible community-dwelling adults recruited and underwent detailed structured interviews for the baseline PHI survey.

The PHI study was approved by the ethics review committee of the National Healthcare Group (NHG) Domain Specific Review Board (Reference Number: 2015/00269). Written informed consent was obtained from all individual participants after they were fully informed of the study objectives and procedures.

### Frailty assessment

Frailty was determined using the revised five-item FRAIL scale (Fatigue, Resistance, Ambulation, Illnesses, & Malnutrition) with the “Malnutrition” replacing the “Loss of weight” in the original FRAIL scale (Fatigue, Resistance, Ambulation, Illnesses, & Loss of weight) [23, 24]. Each item is scored either 0 or 1. The revised FRAIL scale is scored from 0 (best) to 5 (worst) and is translated into three categories: robust (0), pre-frail (1–2), and frail (3–5). Similar to other studies [23, 24], we operationalised the FRAIL scale based on information obtained from specific questions included in the PHI survey questionnaire. “Fatigue” was measured by asking how often they felt tired with responses of “more than half the days” or “nearly every day” scored 1. “Resistance” was assessed by asking their difficulty in walking up and down one flight of stairs without using handrail, and “Ambulation” was measured by asking their difficulty in walking around one floor of home or several blocks without aids; “quite a lot” or “cannot do” responses were each scored as 1. “Illness” was scored 1 for those who reported 5 or more illnesses out of 14 illnesses. “Malnutrition” was scored 1 if Body Mass Index (BMI) < 18.5 or MNA screening score < 8 or Mini Nutritional Assessment (MNA) total score < 17. A complete description of the revised FRAIL scale items’ scoring criteria is provided in Table 1.

**Table 1:** FRAIL scale items

Item	Criteria
Fatigue	1. “Over the last 2 weeks, how often have you been bothered by feeling tired or having little energy?” 0 = Not at all, 1 = Several days, 2 = More than half the days, 3 = Nearly every day Responses of “2” or “3” are scored as 1 and all others as 0. or 2. “Over the last 4 weeks, how often have you been bothered by getting tired very easily?” 0 = Not at all, 1 = Several days, 2 = More than half the days, 3 = Nearly every day Responses of “2” or “3” are scored as 1 and all others as 0.
Resistance	1. Stairs in Activities of Daily Living 1 = Unable to climb stairs, 2 = Assistance is required in all aspects of stair climbing, 3 = Able to ascent/descend but is unable to carry walking aids, and needs supervision and assistance, 4 = Generally no assistance is required, 5 = Able to go up and down a flight of stairs safely without help or supervision Responses of “1”, “2” or “3” are scored as 1 and all others as 0. or 2. “How much difficulty do you have in going up & down a flight of stairs without using handrail?” 5 = None, 4 = A little, 3 = Some, 2 = Quite a lot, 1 = Cannot do Responses of “1” or “2” are scored as 1 and all others as 0.
Ambulation	1. Ambulation in Activities of Daily Living 1 = Dependent in ambulation, 2 = Constant presence of one or more assistants is required during ambulation, 3 = Assistance is required with reaching aids and/or their manipulation. One person is required to offer assistance, 4 = Independent in ambulation but unable to walk 50 yards/metres without help, or supervision is needed for confidence or safety in hazardous situations, 5 = Must be able to use crutches, canes, or a walker, and walk 50 m/yards without help or supervision. Responses of “1”, “2”, “3” or “4” are scored as 1. or 2. “How much difficulty do you have in walking around one floor of your home, taking into consideration thresholds, doors, furniture, and a variety of floor coverings?” 5 = None, 4 = A little, 3 = Some, 2 = Quite a lot, 1 = Cannot do Responses of “1” or “2” are scored as 1 and all others as 0. or 3. “How much difficulty do you have waling several blocks?” 5 = None, 4 = A little, 3 = Some, 2 = Quite a lot, 1 = Cannot do Responses of “1” or “2” are scored as 1 and all others as 0.
Illnesses	“Have you ever been told to have any of these conditions by a Western-trained doctor?” The conditions include diabetes, high blood pressure, high blood cholesterol, heart failure, stroke / transient ischaemic attacks, asthma, chronic bronchitis/ emphysema/COPD, chronic kidney disease, cancer, osteoarthritis/gout/rheumatoid arthritis, osteoporosis, dementia/Alzheimer’s, schizophrenia, Parkinson) 1 = Yes, 0 = No Responses of “1” are scored as 1.
Malnutrition	1. Body Mass Index < 18.5or 2. Screening score of the Mini Nutritional Assessment < 8 or total score < 17

The revised FRAIL scale score ranges from 0 (best) to 5 (worst)

0: Robust, 1–2: Pre-frail, 3–5: Frail

### Healthcare utilisation

The healthcare utilisation data during the retrospective 6-month and prospective 6-month periods were obtained from RHS database [25]. The RHS database contains linked NHG polyclinic visit records, specialist outpatient clinic (SOC) visit records, emergency department (ED) attendance records, day surgery (DS) attendance records and hospital discharge records from three government hospitals - Tan Tock Seng Hospital, Khoo Teck Puat Hospital and Institute of Mental Health, chronic disease management system records and mortality records from local registries. The healthcare utilisation data were categorised according to the main healthcare settings into polyclinic visits, SOC visits, ED visits, DS attendances and hospitalisations. Polyclinic visits refer to doctor consultation and technical visits made by the individual to any of the nine linked NHG polyclinics. SOC visits and ED visits refer to visits to the specialists in outpatient clinics and the emergency rooms located within three government hospitals, respectively. Similarly, DS attendances refer to surgical procedures performed in day surgery rooms where patients were discharged on the same day without admitting to inpatient wards; and hospitalisations refer to inpatient episodes with at least one overnight stay at these hospitals. The survey data and healthcare utilisation data were linked using NRIC numbers which were removed thereafter for data analysis.

### Other variables

We controlled for the confounding effects of covariates to examine the independent effect of frailty on the rates of healthcare utilisation in different care settings. These covariates included demographic factors (age, gender (male / female), Chinese (yes / no), marital status (single / married / widowed or divorced), living arrangement (alone / with others)) [8, 26] and smoking status (non-smoker / past smoker / current smoker) [17]. Highest education level (no formal education / primary / secondary or above) and self-perceived money sufficiency for basic living needs (sufficient / insufficient) were also included as control variables as they are enabling factors which influence individuals’ health seeking behaviours and healthcare utilisation [8, 27, 28].

Multimorbidity and disability, which are related to but also distinct from frailty [29, 30], were commonly adjusted in studies examining the association between frailty and healthcare utilisation [8, 14]. We controlled for multimorbidity as a dichotomous variable (yes / no) which was defined as the presence of two or more of the following 17 chronic conditions: dyslipidemia, high blood pressure, diabetes, chronic kidney disease (CKD), heart attack / ischemic heart disease, heart failure, stroke / transient ischemic attack, asthma, chronic bronchitis / emphysema / chronic obstructive pulmonary disease (COPD), cancer, osteoarthritis / gout / rheumatoid arthritis, osteoporosis, depression, anxiety disorder, schizophrenia, dementia / Alzheimer’s, and Parkinson’s disease [20] (The prevalence of individual chronic diseases among the study participants is presented in the Supplementary Table S1). Disability, which was determined based on whether assistance was required in any of the ten activities of daily living (ADLs) (yes / no) measured using the Modified Barthel Index [31], was also controlled in the models.

### Statistical analysis

Characteristics of the study population were described using mean and standard deviation (SD) for continuous variables, and frequency and percentages for categorical variables. Mean and SD were used to describe healthcare utilisation in every frailty group. To examine the differences in characteristics and utilisation across frailty groups, one-way analysis of covariance (ANOVA) tests (normally distributed) or Kruskal-Wallis H tests (non-normally distributed) were performed for continuous variables, and chi-squared tests were conducted for categorical variables.

Healthcare utilisation by settings are count variables characterised by a point mass at zero followed by a right-skewed, discrete distribution, and non-negative values [32, 33]. Given the over-dispersion of data (the conditional variance is larger than the conditional mean), a negative binomial distribution was chosen over a Poisson regression [34]. Healthcare utilisation in five settings formed five different dependent variables and were analysed independently; and the three-level frailty category (robust, pre-frail, frail) was the independent variable of interest. We further adjusted for control variables including demographic factors (including age, gender, ethnic group, marital status, and living arrangement), socioeconomic status (highest education level, self-perceived money insufficiency), smoking status, multimorbidity, and disability. The results were presented as incidence-rate ratios (IRRs) and their corresponding 95% confidence intervals (CIs). All analyses were performed using Stata/SE 16.1. A *p* value of less than 0.05 was set as the level of significance.

## Results

### Characteristics of study population

Our sample comprised 701 older adults. Their mean age was 70.5 years (SD 8.2). The majority were of Chinese ethnicity (84%), female (57%) and were living with others (81%). The prevalence of multimorbidity and disability (assistance required for any ADLs) among this population was 70 and 15%, respectively. The proportion of pre-frail and frail individuals measured using the revised FRAIL scale was 28 and 7%, respectively (Table 2).

**Table 2:** Characteristics of participants at baseline by frailty groups, n (%)

Characteristics	Overall (***N*** = 701)	Robust (***n*** = 454, 64.8%)	Pre-frail (***n*** = 194, 27.7%)	Frail (***n*** = 53, 7.5%)	***p***-value
**Age, mean ± SD**	70.5 ± 8.2	68.3 ± 6.6	73.1 ± 8.6	79.1 ± 9.8	*< 0.001*
**Female (n, %)**	397 (56.6)	245 (54.0)	122 (62.9)	30 (56.6)	*0.111*
**Chinese**	591 (84.3)	398 (87.7)	153 (78.9)	40 (75.5)	*0.003*
**Marital status**					*0.001*
Single	88 (12.5)	64 (14.1)	21 (10.8)	3 (5.7)	
Married	410 (58.5)	279 (61.5)	107 (55.2)	24 (45.3)	
Divorce/widowed	203 (29.0)	111 (24.4)	66 (34.0)	26 (49.0)	
**Highest education**					*< 0.001*
No formal education	245 (35.0)	123 (27.1)	92 (47.4)	30 (56.6)	
Primary	124 (17.7)	85 (18.7)	30 (15.5)	9 (17.0)	
Secondary or higher	332 (47.4)	246 (54.2)	72 (37.1)	14 (26.4)	
**Living alone**	131 (18.7)	90 (19.8)	36 (18.6)	5 (9.4)	*0.185*
**Self-reported money insufficiency**	113 (16.1)	53 (11.7)	45 (23.2)	15 (28.3)	*< 0.010*
**Smoking status**					*0.047*
Non-smoker	527 (75.2)	349 (76.9)	144 (74.2)	34 (64.2)	
Current smoker	63 (9.0)	41 (9.0)	19 (9.8)	3 (5.7)	
Past smoker	111 (15.8)	64 (14.1)	31 (16.0)	16 (30.2)	
**Multimorbidity**	487 (69.5)	276 (60.8)	161 (83.0)	50 (94.3)	*< 0.001*
**Disability**	107 (15.3)	9 (2.0)	58 (29.9)	40 (75.5)	*< 0.001*

The percentages were reflected as column percentages

Comparing the profile across the three frailty groups (Table 2), we showed that frail elderly were significantly older, and had a higher proportion with multimorbidity or required assistance in any ADLs. A significantly lower proportion of frail elderly were single, of Chinese ethnicity; and a higher proportion had no formal education and perceived that they had insufficiency financial means for their daily needs.

### Association between frailty and healthcare utilisation

#### Healthcare utilisation in 6-month period prior to the baseline

Compared to older adults in robust health, significantly higher proportions of SOC and ED visits, DS attendances as well as hospitalisations were observed in those who were pre-frail and frail (Table 3). The mean number of SOC visits, ED visits and hospitalisations also increased corresponding with the increase in frailty levels (all *p* < 0.001). Pre-frail older adults were the dominant users of the polyclinic services with about 42% having polyclinic visits.

**Table 3:** Associations between frailty and healthcare utilisation in different settings during 6-month period prior to the baseline

Healthcare utilisation by setting	Frailty	Yes, n (%)	Mean ± SD	Adjusted IRR^**c**^ (95% CI)
**Polyclinic visits**	**Robust (*n*** **= 454)**	148 (32.6)	0.95 ± 1.96	1.00
	**Pre-frail (*n*** **= 194)**	82 (42.3)	1.47 ± 2.69	1.35 (0.96, 1.91)
	**Frail (*n*** **= 53)**	20 (37.7)	1.26 ± 2.03	1.11 (0.58, 2.10)
	*p-value*	*0.060*^*a*^	*0.024*^*b*^	
**Specialist outpatient clinic visits**	**Robust (*n*** **= 454)**	126 (27.8)	1.22 ± 2.93	1.00
	**Pre-frail (*n*** **= 194)**	79 (40.7)	2.40 ± 5.83	1.65 (1.04, 2.63)
	**Frail (*n*** **= 53)**	27 (50.9)	3.92 ± 5.39	2.82 (1.22, 6.50)
	*p-value*	*< 0.001*	*< 0.001*	
**Emergency department visits**	**Robust (*n*** **= 454)**	28 (6.2)	0.09 ± 0.41	1.00
	**Pre-frail (*n*** **= 194)**	22 (11.3)	0.18 ± 0.63	1.10 (0.55, 2.21)
	**Frail (*n*** **= 53)**	16 (30.2)	0.57 ± 1.01	3.05 (1.14, 8.12)
	*p-value*	*< 0.001*	*< 0.001*	
**Day surgery attendances**	**Robust (*n*** **= 454)**	18 (4.0)	0.06 ± 0.37	1.00
	**Pre-frail (*n*** **= 194)**	10 (5.2)	0.09 ± 0.61	2.02 (0.77, 5.27)
	**Frail (*n*** **= 53)**	6 (11.3)	0.13 ± 0.39	6.41 (1.33, 30.92)
	*p-value*	*0.060*	*0.062*	
**Hospitalisations**	**Robust (*n*** **= 454)**	13 (2.9)	0.04 ± 0.27	1.00
	**Pre-frail (*n*** **= 194)**	20 (10.3)	0.14 ± 0.47	2.06 (0.91, 4.67)
	**Frail (*n*** **= 53)**	15 (28.3)	0.51 ± 0.95	6.72 (2.14, 21.11)
	*p-value*	*< 0.001*	*< 0.001*	

^a^*p*-values were obtained by chi-squared tests

^b^*p*-values were obtained by Kruskal-Wallis H tests

^c^IRR: Incidence rate ratio. Adjusted for age, female, Chinese, marital status, highest education level, living alone, self-reported money insufficiency, smoking status, multimorbidity, and any assistance required in ADLs

After adjusting for all covariates including multimorbidity and disability, the negative binomial regression results showed that frailty was statistically associated with the adjusted rates of SOC visits, ED visits, DS attendances and hospitalisations in 6-month period prior to the baseline. Relative to the robust group, individuals who were frail had 2.8 times the rate of SOC visits; had 3.1 times the rate of ED visits; and had a rate 6.4 times and 6.7 times greater for DS attendances and hospitalisations respectively (Table 3). Pre-frail individuals had 1.7 times and 2.1 times the rate of SOC visits and hospitalisations respectively compared to their robust counterpart.

#### Healthcare utilisation during 6-month period after the baseline

Similarly, during the 6-month period after the baseline, significantly higher proportion and mean number of SOC and ED visits, as well as hospitalisations in pre-frail and frail older adults were observed compared to their robust peers (Table 4). After adjusted for all covariates, frail older adults had 3.3 times the rate of SOC visits, 6.4 times the rate of ED visits, and 5.8 times and 13.1 times the rate of DS attendances and hospitalisations respectively compared to their robust counterpart during the 6-month period after the baseline. No significant difference in rate was observed for polyclinic visits. Pre-frail individuals had 1.5 times, 2.6 times and 3.8 times higher rate of polyclinic visits, ED visits and hospitalisations respectively compared to their robust counterpart (Table 4).

**Table 4:** Associations between frailty and healthcare utilisation in different settings during the 6-month period after the baseline

Healthcare utilisation by setting	Frailty	Yes, n (%)	Mean ± SD	Adjusted IRR^**c**^ (95% CI)
**Polyclinic visits**	**Robust (*n*** **= 454)**	153 (33.7)	0.97 ± 2.3	1.00
	**Pre-frail (*n*** **= 194)**	82 (42.3)	1.64 ± 4.18	1.54 (1.08, 2.19)
	**Frail (*n*** **= 53)**	20 (37.7)	1.11 ± 1.82	1.17 (0.60, 2.29)
	*p-value*	*0.113*^*a*^	*0.080*^*b*^	
**Specialist outpatient clinic visits**	**Robust (*n*** **= 454)**	139 (30.6)	1.21 ± 2.61	1.00
	**Pre-frail (*n*** **= 194)**	70 (36.1)	2.03 ± 4.13	1.48 (0.96, 2.27)
	**Frail (*n*** **= 53)**	31 (58.5)	5.08 ± 7.32	3.31 (1.56, 7.06)
	*p-value*	*< 0.001*	*< 0.001*	
**Emergency department visits**	**Robust (*n*** **= 454)**	20 (4.4)	0.05 ± 0.25	1.00
	**Pre-frail (*n*** **= 194)**	20 (10.3)	0.19 ± 0.73	2.55 (1.25, 5.20)
	**Frail (*n*** **= 53)**	16 (30.2)	0.47 ± 0.82	6.40 (2.38, 17.24)
	*p-value*	*< 0.001*	*< 0.001*	
**Day surgery attendances**	**Robust (*n*** **= 454)**	24 (5.3)	0.06 ± 0.31	1.00
	**Pre-frail (*n*** **= 194)**	11 (5.7)	0.09 ± 0.47	1.77 (0.77, 4.06)
	**Frail (*n*** **= 53)**	5 (9.4)	0.13 ± 0.44	5.75 (1.28, 25.78)
	*p-value*	*0.468*	*0.450*	
**Hospitalisations**	**Robust (*n*** **= 454)**	11 (2.4)	0.03 ± 0.17	1.00
	**Pre-frail (*n*** **= 194)**	19 (9.8)	0.12 ± 0.41	3.76 (1.66, 8.53)
	**Frail (*n*** **= 53)**	16 (30.2)	0.53 ± 0.97	13.11 (4.90, 35.04)
	*p-value*	*< 0.001*	*< 0.001*	

^a^*p*-values were obtained by chi-squared tests

^b^*p*-values were obtained by Kruskal-Wallis H tests

^c^IRR: Incidence rate ratio. Adjusted for age, female, Chinese, marital status, highest education level, living alone, self-reported money insufficiency, smoking status, multimorbidity, and any assistance required in ADLs

## Discussion

We examined the associations between frailty and healthcare utilisation in different public healthcare settings among community-dwelling adults aged 60 years and above in Singapore. Although the issue of frailty is predominant among people aged 70 years and above, the public health system in Singapore proactively puts in efforts and resources in identifying the pre-frail individuals and those at risk of frailty, and extends services to people who are aged 60 years and above when designing programmes and interventions. As such, we included participants aged 60 years and above as the study population. The results showed that the association between frailty and healthcare utilisation varied in different settings. While the frail elderly in the community had significantly higher proportion and number of SOC visits, ED visits, day surgery attendances and hospitalisations in the 6-month period prior to and after the baseline, their utilisation of public primary care services was lower relative to their pre-frail or robust peers.

Prior studies consistently reported that increasing frailty is associated with substantial increase in hospital admissions, measured either retrospectively or prospectively [8, 16, 35]. We also observed that the frail older adults in the study had more hospitalisations than their robust and pre-frail peers, regardless whether the hospitalisations occurred prior to or after the baseline. Their association is persistent even after adjusting for the socio-demographics, multimorbidity and disability. Among the healthcare service utilisation in the five different care settings, our study found that frailty had the most significant impact on hospitalisations in both 6-month period prior to and after the baseline, which is consistent with findings reported in prior studies [8, 12, 36]. The association between frailty and hospitalisations indicates that frail elderly in Singapore tend to present to the healthcare system, especially tertiary care, when they are in a more severe stage of frailty [37].

Unlike prior studies which reported that frailty had a positive association with probability of use of primary care services in general practitioner clinics [8, 15, 16, 36], our study found frail individuals did not have higher risk of utilising more polyclinic services than their robust counterparts. Instead, the older adults who were in the pre-frail stage tended to use more polyclinic services. This suggests that when older adults deteriorate from robust health stage to pre-frail stage, their use of primary care services increase significantly; and when older adults are in a more severe stage of frailty, their needs may shift towards increased specialist care services. However, the results should be interpreted with caution as only about 20% of the total primary care services in Singapore are provided by polyclinics and the left 80% are provided by private general practitioners (GPs). Although polyclinics provide a relatively larger percentage of care for patients with chronic and more complex conditions, the omission of GP utilisation data in the RHS database makes it challenging to infer the association between frailty and the total primary care utilisation.

Although the association between frailty and healthcare utilisation of specialist outpatient care is less investigated compared with that of inpatient services, prior studies do suggest that frailty has positive association with the use of specialist outpatient services [8, 38]. Our study provides additional support for their association, regardless whether the SOC visits occurred in 6 months prior to or after the baseline. This reflects that an increase in the degree of frailty among older adults corresponds with a greater need for comprehensive and specialised health care services [38, 39].

The association between frailty and number of surgical procedures performed in DS rooms were scarcely examined separately in prior studies. A relatively higher proportion and rate of DS attendances among frail individuals was observed in the study, with no significant difference in associations between 6 months prior to and after the baseline. However, we are unable to draw any causal conclusion between frailty and DS attendances. Although the degree of frailty prior to general surgery is increasingly used for risk-stratification, as most of the DS procedures are considered low-stress procedures for older adults, the findings in the study suggests that frail individuals have a greater need for day surgeries. As pre-operative frailty is associated with adverse clinical outcomes even for low-stress procedures [40], comprehensive frailty screening should be conducted prior to surgical procedures not only for optimization before surgery but also for targeted interventions before and after surgery.

It has been well established that multimorbidity is bidirectionally associated with frailty [41] and greater healthcare utilisation [42]. A recent study suggests that frailty may be directly related to kidney function [43]. We also observed that CKD, which accounted for 20% of the multimorbidity, was also highly prevalent among frail groups (49%). Our result showed consistent associations between multimorbidity and number of polyclinics and SOC visits in both 6-month period prior to and after the baseline. However, the associations between multimorbidity and number of ED and DS attendances observed in these two periods were controversial, and no association was observed between multimorbidity and hospitalisation. This could probably be explained by the prevalence of individual chronic conditions that were used to define multimorbidity. As dyslipidaemia and high blood pressure were the most prevalent chronic conditions among the study population with prevalence of 66 and 64% respectively, the multimorbidity was highly attributed to either of the two conditions (high blood pressure 84% and dyslipidaemia 88%), which are usually monitored in primary care settings or SOCs. While polytherapy is often mandatory in the management of multimorbidity [44], therapeutic decisions for elderly people should be approached cautiously as a more elevated polytherapy index is associated with the development of frailty [45] and increased healthcare utilisation.

### Strengths and limitations

To the best of our knowledge, this is the first study investigating the association between frailty and patterns of healthcare utilisation in different care settings in Singapore. We examined the association using both retrospective and prospective utilisation data and found consistent relationship between frailty and healthcare utilisation in respective settings. However, as there is still some uncertainty whether frailty occurred before or after healthcare utilisation, the claim of causal inferences is limited.

The analyses presented in the study used number of hospitalisations to capture the inpatient utilisation. It is acknowledged that number of hospitalisations is a partial indicator of inpatient utilisation, length of stay, which also reflects another important aspect of inpatient utilisation [46], was not measured.

The healthcare utilisation data were derived from one RHS database, as such, healthcare utilisation in other RHS, GPs, private SOCs and hospitals, as well as home care services provided by Voluntary Welfare Organisations were not included. This may cause under-estimation of the association between frailty and healthcare utilisation. However, as the participants were the residents in the region with their health entrusted to the respective RHS, the majority of their utilisation in public healthcare services should have been captured and serve the purpose of understanding the patterns of service delivery in these five care settings for older people with different frailty status within the defined geographical region served by the RHS.

## Conclusions

Frailty was positively associated with SOC visits, ED visits, DS attendances and hospitalisations during 6-month period prior to or after the baseline among community-dwelling older adults. Frail individuals tended to have higher rate of SOC and ED visits, DS attendances and hospitalisations compared to their robust counterpart. As frailty is a potentially reversible health state with early screening and intervention, identifying the pre-frail and frail elderly in the community, and providing effective interventions at early stage could be an effective strategy of reducing or delaying utilisation of secondary and tertiary care services.

## Supplementary information

**Additional file 1.**

## Data Availability

The datasets used and/or analysed during the current study are available from the corresponding author on reasonable request.

## Abbreviations

ADLs: Activities of daily living
ANOVA: Analysis of covariance
BMI: Body mass index
CI: Confidence interval
CKD: Chronic kidney disease
COPD: Chronic obstructive pulmonary disease
DS: Day surgery
ED: Emergency departments
GPs: General practitioners
IRR: Incidence rate ratio
MNA: Mini Nutritional Assessment
NHG: National Healthcare Group
NRIC: National Registration Identity Card
OR: Odds ratio
PHI: Population Health Index
RHS: Regional healthcare system
SD: Standard deviation
SOC: Specialist outpatient clinic
