# Supplementary material for: Frailty and healthcare utilisation across care settings among community-dwelling older adults in Singapore
Source: BMC Geriatr. 2020 Oct 6;20:389. doi: 10.1186/s12877-020-01800-8 (PMC7542115; doi:10.1186/s12877-020-01800-8)
Supplement: Supplementary file 1 — Additional file 1. [file 12877_2020_1800_MOESM1_ESM.docx]

Table S1. Prevalence of chronic conditions by Frailty group, n (%)

| Chronic conditions | Overall (N=701) | Robust (n=454) | Prefrail (n=194) | Frail (n=53) | p-value |
| --- | --- | --- | --- | --- | --- |
| Dyslipidaemia | 460 (65.6) | 273 (60.1) | 143 (73.7) | 44 (83.0) | .000 |
| High blood pressure | 445 (63.5) | 254 (55.9) | 145 (74.7) | 46 (86.8) | .000 |
| Osteoarthritis / Gout / Rheumatoid | 221 (31.5) | 101 (22.2) | 90 (46.4) | 30 (56.6) | .000 |
| Diabetes | 204 (29.1) | 103 (22.7) | 76 (39.2) | 25 (47.2) | .000 |
| Chronic Kidney disease | 96 (13.7) | 33 (7.3) | 37 (19.1) | 26 (49.1) | .000 |
| Osteoporosis | 91 (13.0) | 34 (7.5) | 37 (19.1) | 20 (37.7) | .000 |
| Heart Attack | 77 (11.0) | 37 (8.1) | 29 (14.9) | 11 (20.8) | .002 |
| Stroke / transient ischemia attack | 75 (10.7) | 29 (6.4) | 24 (12.4) | 22 (41.5) | .000 |
| Cancer | 62 (8.8) | 25 (5.5) | 24 (12.4) | 13 (24.5) | .000 |
| Heart Failure | 43 (6.1) | 14 (3.1) | 13 (6.7) | 16 (30.2) | .000 |
| Asthma | 34 (4.9) | 18 (4.0) | 10 (5.2) | 6 (11.3) | .060 |
| Depression | 28 (4.0) | 9 (2.0) | 12 (6.2) | 7 (13.2) | .000 |
| Chronic bronchitis / emphysema / COPD | 26 (3.7) | 8 (1.8) | 11 (5.7) | 7 (13.2) | .000 |
| Anxiety | 25 (3.6) | 17 (3.7) | 3 (1.5) | 5 (9.4) | .022 |
| Dementia /Alzheimer | 18 (2.6) | 4 (0.9) | 7 (3.6) | 7 (13.2) | .000 |
| Schizophrenia | 9 (1.3) | 5 (1.1) | 2 (1.0) | 2 (3.8) | .245 |
| Parkinson disease | 9 (1.3) | 2 (0.4) | 4 (2.1) | 3 (5.7) | .003 |

*The percentages were reflected as column percentages.*
